# Green Technology Fitness

**DOI:** 10.3390/e20100776

**Published:** 2018-10-10

**Authors:** Angelica Sbardella, François Perruchas, Lorenzo Napolitano, Nicolò Barbieri, Davide Consoli

**Affiliations:** 1 Department of Economics and Finance, University of Rome Tor Vergata, 00133 Rome, Italy; 2 INGENIO (CSIC-Universitat Politecnica de Valencia), 46022 Valencia, Spain; 3 Institute for Complex Systems, National Research Council, 00185 Rome, Italy; 4 Department of Economics and Management, University of Ferrara, 44121 Ferrara, Italy

**Keywords:** green technology, fitness, capabilities, economic development

## Abstract

The present study provides an analysis of empirical regularities in the development of green technology. We use patent data to examine inventions that can be traced to the environment-related catalogue (ENV-Tech) covering technologies in environmental management, water-related adaptation and climate change mitigation. Furthermore, we employ the Economic Fitness-Complexity (EFC) approach to assess their development and geographical distribution across countries between 1970 and 2010. This allows us to identify three typologies of countries: leaders, laggards and catch-up. While, as expected, there is a direct relationship between GDP per capita and invention capacity, we also document the remarkable growth of East Asia countries that started from the periphery and rapidly established themselves as key actors. This geographical pattern coincides with higher integration across domains so that, while the relative development of individual areas may have peaked, there is now demand for greater interoperability across green technologies.

## 1. Introduction

There is broad consensus among academics and policy makers that accelerating the development of new low-carbon technologies and promoting their global application are crucial steps, albeit not the only ones, towards containing and preventing greenhouse gas (GHG) emissions. To be sure, climate change is a global phenomenon with marked local manifestations, which implies that geographical areas differ significantly both in their exposure as well as in their ability to respond effectively to climate events. Indeed the striking paradox is that while environmentally friendly technologies emerge primarily in industrialised countries, the urgency to mitigate GHG emissions is stronger in emerging economies. Last but not least, besides the traditional negative externalities due to non-appropriability and non-exclusivity of knowledge, green technologies engender also positive externalities in the form of improvements to the quality of the environment.

These features highlight the importance of institutional conditions for promoting or thwarting sustainable economic growth. Governance mechanisms that are crucial to create incentives for efficient use of natural resources and for environmental conservation, while minimizing the prospect of market failures, are spatially bound [1]. Spatial features are also relevant because the generation and diffusion of knowledge stem from the recombination of ideas [2,3] among agents that have limited access to information, as well as imperfect capacity to absorb, process, and respond to it [4]. Because information exchange entails costs that increase with the diversity of the attendant knowledge base, higher coherence between activities is expected to facilitate the likelihood of innovation [5,6,7].

The key point is that economic development builds on existing local capabilities to generate distinctive technological and industrial profiles [8,9]. A major driver of the distinctiveness of these trajectories is indeed the composition of knowledge, that is, the number of underlying inputs and the interdependence between them [10,11,12]. The greater and more diverse the spectrum of know-how, the more complex the domains to which this knowledge is applied, be they products [13,14], industries [15] or technologies [16]. Empirical evidence provides clear indications about these patterns. First, there are significant differences in the complexity of knowledge produced across geographical locations. Second, only a few areas exhibit proficiency in complex activities, and this usually correlates with their long-run economic development. However, by virtue of path-dependence, while investing in complex technologies is beneficial in principle, many areas simply lack the necessary competences and, most fundamentally, their underlying conditions prevent them from creating a new path of development. As a consequence, and third, these features are dynamically self-reinforcing.

In this paper, we employ analytic techniques developed within the Economic Fitness-Complexity (EFC) approach to economic prediction [17] in order to assess the development and geographical distribution of green technologies between 1970 and 2010. EFC is a data-driven methodology that originally targeted the relation between the composition of the export baskets of countries and their potential to become more developed economies. The idea behind this methodology is that for a country to become competitive in the production of a given good, it must first acquire the necessary skills. However, the process leading to the acquisition of new capabilities is by its very nature cumulative and highly path-dependent, which is consistent with the fundamental intuition that *complex* products requiring advanced skills will be exported mostly, if not only, by *high fitness* countries that will also be competitive in the production and trade of less complex goods. Capabilities are generally not observable, and can be conceived as a latent intermediate layer between countries and products in an ideal tri-partite network. Some recent successful applications of EFC [18,19] have aimed to extract information about the effects of accumulated capabilities by studying the bipartite network of countries and exported goods. These studies have shown that the EFC algorithm has considerable predictive power of the future development of countries, as measured by their future per capita GDP. Among its outputs, the algorithm features a ranking of country fitness values that proxy how advanced the set of capabilities of each country is, and a ranking of product complexity values that proxies how advanced are the capabilities required to produce each product. The satisfying performance of the method on empirical data has also led to the development of a diversified array of methods and indicators that rest on the same premises. One such derived measure, which is called *sector fitness*, is a straightforward modification of the method proposed by [17]. This narrows down the analysis to a set of similarly classified products and generates a snapshot of the strength of each country in a specific sector of activity. Notice that the EFC method exhibits substantial versatility. For instance, it has been applied successfully to study labour sectors instead of exported products [20]; another recent application of directly related techniques has been employed to analyse the capability spillovers between the patenting activity, the scientific production and the export profiles of countries [21].

For this study, we use patent applications as a proxy of capability. The main source is the European Patent Office (EPO) Worldwide Patent Statistical Database (Patstat) containing patent applications that can be traced to the environment-related technologies catalogue (Env-Tech) developed by the Organisation for Economic Co-operation and Development (OECD) [22] and organised in macro-domains such as environmental management, water-related adaptation, and climate change mitigation. The transliteration of the EFC approach to this hitherto unexplored empirical context rests on the idea that the criteria for assigning patent applications to specific domains (i.e., technological classes) are identifying characteristics of the expertise that is necessary for successful invention. In particular, the co-occurrence of technological classes in a country allows us to identify the extent to which inventions and the attending capabilities are common across countries. Accordingly, a country that has a diversified portfolio of technologies spanning from the most to the least complex ones will have higher fitness while, in turn, complex technologies appear almost exclusively in the portfolio of high-fitness countries. As a consequence, more specialised (or less diversified) countries operate almost exclusively in less complex sectors. In other words, the portfolio of activities of low-fitness countries is (almost) nested in that of higher-fitness countries.

Bearing in mind the benefits and the shortcomings of using patent data for the study of technology development (see e.g., [23,24,25]), the juxtaposition of the above database and methodology yields proxies of environment-related inventive activities that allow cross-country and cross-technology comparisons. In particular, the set of indicators proposed here informs a ranking of countries propensity to create new green technology as well as of the development these technologies. While we remain agnostic about the pathways through which countries develop and apply capabilities to environmental issues, we provide insights into the extent to which each country contributes to the global network of technological capabilities, as well as into the extent to which the technologies grow and develop as a result of distributed inventive efforts. Furthermore, we expect that a thorough mapping of *who* is inventing and *in what* can enrich the current debate on leaders and laggards in the transition to sustainable societies. A detailed analysis of the contextual institutional processes that shape the accumulation of innovative competences within countries—such as i.e., research and development, labour markets, etc.—and how this affects differential performance between countries is beyond the scope of the current study, and is left for future research.

## 2. Materials and Methods

### 2.1. Data

The main data source is the Patstat database [26] of patent applications. In particular, we exploit patent classification codes to identify inventions in the domain of environment-friendly technologies within the classification Env-Tech elaborated by the OECD [22], which groups International Patent Classification (IPC) and Cooperative Patent Classification (CPC) codes into 94 green technologies. The IPC and CPC are two widespread technology classification systems employed by patent offices to classify the patent documents based on the technological areas in which they claim to be novel. Both systems exhibit a hierarchical structure that describes the technical content of the patents in progressively finer detail at lower levels of aggregation [27].

We also exploit information in Patstat about patent families—i.e., collections of patents that can be linked to one or more common ‘ancestor’ patent documents. These collections typically contain documents relating to the multiple applications involved in protecting the same inventions in multiple countries, and are our unit of analysis [28]. We identify 1,179,657 patent families (or 2,690,606 patent applications) to which at least one Env-Tech classification code is assigned. The resulting data set includes patent families, filed between 1970 and 2010, concerning a large share of green technologies in the following fields (and the associated 1-digit Env-Tech code):

- environmental management (1)
- water management (2)
- climate-change mitigation technologies (CCMTs) related to energy production (4)
- capture and storage of greenhouse gases (5)
- CCMTs related to transportation (6)
- CCMTs related to buildings (7)
- CCMTs related to waste-water and waste management (8)
- CCMTs in the production of goods (9)

**Figure 1:**
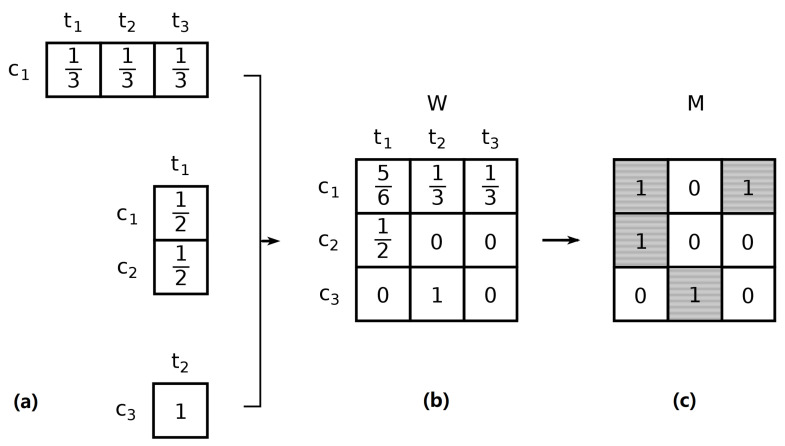
Example of data construction. (**a**) Assume that the there are only three patent families to account for in a given period *y*: one (**top**) developed in a single country $c_{1}$ that innovates in three distinct fields ($t_{1}$, $t_{2}$, and $t_{3}$); another one (**centre**) developed by inventors residing in two countries that innovates in a single technology; and (**bottom**) a single-country, single-technology patent family. All patents are attributed equal weights and the attribution to country-technology pairs is fractional. (**b**) The union of the country-technology combinations of all inventions is combined into the weighted matrix W(y). (**c**) W(y) is binary to reflect revealed comparative advantage yielding M(y), which is the input of the EFC algorithm.

To measure national knowledge bases, we assign patent applications to countries using the inventor’s address information in Patstat. This procedure yields a weighted matrix W(y) in which each element $W_{c,t}\left(y\right)$ represents the fractional count of inventions attributed to country *c* and technology *t* in year *y* (see Figure 1 for a detailed example). Such a value can be considered a proxy for the degree of involvement in green technology *t* of inventors residing in *c* (see Appendix C for a more detailed description of the procedure and the data sources).

### 2.2. A *Fitness Approach* to Green Technology

As mentioned in Section 1, we focus on the *green sector-fitness* of countries that host inventors of green technologies and the complexity of the green technology classes included in the inventions. Recall that the peculiarity of *sector-fitness* lies therein, to compute it, we do not extract information from the whole technology spectrum (all possible IPC and/or CPC classes) but, rather, we restrain to a subset of classes that identify the relevant area for the study of a particular sector of activity, in our case, green technologies. Furthermore, recall that this approach has already been employed successfully in the study of country exports to break down the fitness profile into individual industries. No doubt, applying *sector-fitness* to technologies does imply some risks. The main issue is that the interpretation of the sector fitness might not be as straightforward for technologies as it is for industries. In fact, defining an industrial sector from an aggregation of products implies grouping together objects that are classified unequivocally and generally assigned to only one sector. The same cannot be said for technologies, since multiple technological fields, namely the objects that we use to define the technological equivalent of a sector, usually contribute to the same patent, and these fields tend to be quite distant within the classification tree. For this reason, studying green technology classes in isolation neglects a wealth of non-green classes that however are part of green inventions. Bearing in mind these caveats, we expect that the selection of the data involved in applying the sector fitness approach to studying green technologies still yields reasonable results. The interested reader is referred to Appendix B for a more detailed discussion.

Computations involve EFC algorithm wherein inputs are binary matrices of countries (rows) and classes (columns). The underlying assumption is that each patent family weights one unit which is shared between (country, class) pairs. Since patent applications can be unambiguously attributed to their filing year, it is natural to build a series of yearly weighted matrices W(y), where each matrix element $W_{c,t}\left(y\right)$ is the sum of the shares of applications filed in year *y* that can be traced back to country *c* and green-technology class *t*. The EFC algorithm requires a binary matrix as input, thus, for each year *y*, we binarize W(y) based on Revealed Comparative Advantage [29,30] and obtain M(y) such that:

(1) $$M_{c,t}\left(y\right)=\left\{\begin{array}{cc} 1 & \mathrm{if}\;\frac{W_{c,t}}{\sum_{t^{\prime }}W_{c,t^{\prime }}}>\frac{\sum_{c^{\prime }}W_{c^{\prime },t}}{\sum_{c^{\prime },t^{\prime }}Wc^{\prime },t^{\prime }}\\ 0 & \mathrm{otherwise}. \end{array}\right.$$

The binary matrices are then fed to the EFC algorithm to yield non-negative scores and rankings for fitness as well as complexity. In formulae:

(2) $$\begin{array}{c} \left\{\begin{array}{cc} {\tilde{F}}_{c}^{\left(n\right)}=\sum_{t}M_{c,t}Q_{t}^{\left(n-1\right)}, & Q_{t}^{\left(n\right)}=\frac{{\tilde{Q}}_{t}^{\left(n\right)}}{<{\tilde{Q}}_{t}^{\left(n\right)}>}\\ {\tilde{Q}}_{t}^{\left(n\right)}=\frac{1}{\sum_{c}M_{c,t}\frac{1}{F_{c}^{\left(n\right)}}}, & F_{c}^{\left(n\right)}=\frac{{\tilde{F}}_{c}^{\left(n\right)}}{<{\tilde{F}}_{c}^{\left(n\right)}>} \end{array}\right. \end{array}$$

with initial condition:

(3) $$\sum_{t}Q_{t}^{\left(0\right)}=1\;\forall t.$$

The fitness of a country is thus defined as the average complexity of its technologies. The definition of the complexity of a technology, instead, involves a non-linear equation that attributes lower complexity to the technologies patented by low-fitness countries. It should be noted that, depending on the structure of M(y), the scores of the lower-ranked entities can converge to zero [31]. Fortunately, rankings remain consistent and can therefore be trusted. For this reason, focusing on country and technology rankings is a good strategy.

It is worth mentioning that patenting intensity (and coverage) in several countries has grown sharply in the past decades. Moreover, filing of new patent applications in specific technological areas is relatively intermittent, meaning that for a given pair (c,t), the corresponding cell in matrix M(y) is often different from that in M(y+1). This is more apparent if technological codes are disaggregated, and can induce some noise. A possible solution is to give up details for inter-temporal stability by aggregating Env-Tech technology classes from 3 to 2 digits [32]. A further complementary approach entails averaging over multiple yearly snapshots of W(t)

(4) $$W\left(y,\delta \right)=\frac{1}{\delta }\sum_{t=0}^{\delta -1}W\left(y-t\right)$$

before bin arising to obtain M(y,δ). For our analysis, we choose δ=10 and divide the data into four non-overlapping windows—1971–1980, 1981–1990, 1991–2000, and 2000–2010—each labeled using the latest included year (e.g., 2010 stands for the period 2000–2010). Unless otherwise stated, 2-digit technology classes are employed throughout. The interested reader is referred to Appendix D for a more detailed account of the trade-off implied by inter-temporal and technological aggregation of the data as well as the trade-off implied by the choice of the extremes of the time interval included in the analysis.

## 3. Results and Discussion

### 3.1. Green Fitness Ranking: Countries and Technologies

**Figure 2:**
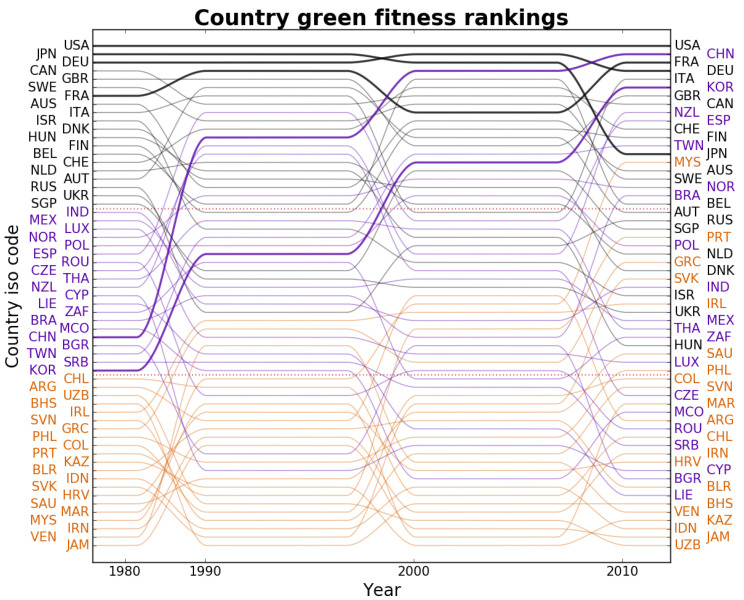
Time evolution of the green fitness ranking of countries from 1980 to 2010. The country labels on the left and right vertical axes are listed from bottom to top in order of increasing fitness in the first and last period of analysis respectively. The lines trace the changes in ranking of each country across decades. Label and line colours refer to the position of countries in the initial ranking: black, violet and purple are associated respectively to the top-, middle-, and bottom-third of the 1980 green fitness ranking. Colours are mixed in 2010, meaning that positions in the ranking have changed substantially for several countries (see e.g., the constant growth of China and South Korea highlighted by the thicker purple lines). The names of the countries associated to the abbreviations reported on the *y*-axis of the plot are reported in Table A2 of Appendix A.

Figure 2 shows the green fitness rankings of all countries across all four time windows. The higher the ranking the more complex the country’s portfolio of green technologies and, thus, the more advanced the invention competences. We provide a synthetic sketch focus on how countries’ innovation capacity evolves over time using colour coding to distinguish three groups depending on the initial ranking: leaders (black), followers (purple), and laggards (orange). To begin with, most of the countries that were leaders in 1980 are still in the top ranking in 2010. Even so, we observe some heterogeneity in their long-term paths. A first group of global leaders such as the United States (USA), France (FRA), Germany (DEU) (in black in Figure 2) maintained a steady high ranking throughout the period, while others—e.g., Japan (JPN), Sweden (SWE), India (IND)—remained mostly in the upper echelons, but also declined slightly and were caught up with in the ranking by some follower and laggard countries. Among these, it is worth mentioning some fast-growing countries, listed by increase in the green fitness ranking, such as Malaysia (MYS), South Korea (KOR), China (CHN), Slovakia (SVK), Portugal (PRT) and Saudi Arabia (SAU). These all started from mid-to bottom positions in 1980 and after an impressive, and steady, acceleration have reached the top part of the ranking. Notice that over time the geographical distribution of inventive activity spreads out, primarily towards Asia, while the presence of Latin American and African countries is only marginal. As regards Europe, the distinction between leaders and followers resonates with the differences between countries in the core and those in the periphery. Notice also that laggard countries exhibit similar stability to leaders, meaning that countries starting in such groups in the 1980 time window tend, with some notable exceptions, to remain in the same group throughout.

**Figure 3:**
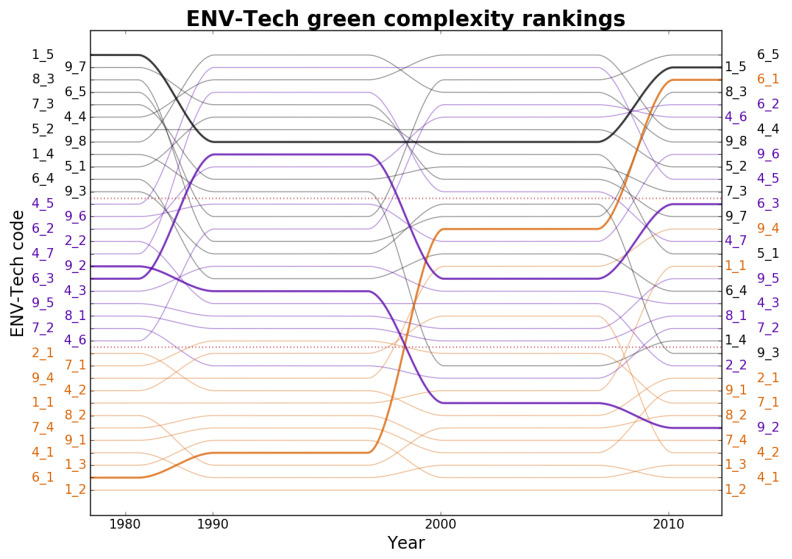
Time evolution of green complexity ranking of ENV-Tech technologies from 1980 to 2010. The technology labels on the left and right vertical axes are listed from bottom to top in order of increasing complexity in the first and last period of analysis respectively. The lines trace the changes in ranking of each technology across decades. Label and line colours refer to the position of technologies in the initial ranking: black, violet and purple are associated respectively to the top-, middle-, and bottom-third of the 1980 green complexity ranking. Colours are mixed in 2010, meaning that positions in the ranking have changed substantially for several technologies. For instance, notice the constant growth of the ENV-Tech technology ‘Road Transport’ (6_1), and the steady decline of the ENV-Tech technology ‘Technologies Relating to Chemical Industry’ (9_2), highlighted respectively by a thicker orange and purple line. The definitions of the technological codes associated to the abbreviations reported on the y-axis of the plot are reported in Table A1 of Appendix A.

Figure 3 lists the Green Complexity of 2-digit environmental technologies in our database and the associated ranking. Again, the idea is that a higher complexity ranking indicates that a technology entails a more advanced array of capabilities. Compared to countries, green technologies exhibit more fluidity, at least in the bottom half of the list, as about half of them have at least one appearance in the top 10 (conversely, only 4 have been in the the top 5). Looking more in detail, three groups of green technologies emerge. The first cluster comprises technologies that consistently rank highest (black in Figure 3), namely ‘Nuclear Energy’ (4_4), ‘Environmental Monitoring’ (1_5), ‘Enabling Technologies for GHG Emissions Mitigation’ (8_3) and ‘Enabling Technologies in Transport’ (6_5)—in fact, each one of them has been top of the list in the period under analysis. In the second cluster (purple in Figure 3) are technologies that, while being consistently high ranking, have at least once slipped out of the top 10. Among these we observe a variety of patterns, some stable technologies—such as ‘Capture or Disposal of Greenhouse Gases other than $CO_{2}$’ (5_2)—some oscillating technologies—such as ‘Technologies for Efficient Electrical Power Generation, Transmission or Distribution’ (4_5), ‘$CO_{2}$ Capture or Storage’ (5_1) or ‘Air Transport’ (6_3)—as well as steady growers—i.e., ‘Road Transport’ (6_1), ‘Rail Transport’ (6_2), ‘Enabling Technologies’ (4_6)—and steady decliners – like ‘Technologies Relating to Chemical Industry’ (9_2) and ‘Climate Change Mitigation Technologies for Sector-Wide Applications’ (9_7). The third cluster (orange in Figure 3) contains technologies that have only been in the top 10 once, e.g., ‘Water Pollution Abatement’ (1_2) and ‘Renewable Energy Generation’ (4_1).

Again, a closer look indicates heterogeneity of patterns over time: the most notable are the ascent of ‘Road Transport’ (6_1) and ‘Technologies in the Production Process for Final Industrial or Consumer Products’ (9_6) in contrast with the decline of ‘Soil Remediation’ (1_4) and ‘Architectural or Constructional Elements Improving the Thermal Performance of Buildings’ (7_3). An interesting indication is that Mitigation technologies rank in general higher than Adaptation. Another notable feature is that almost all Enabling Environmental Technologies—that is, horizontal technologies with potential applicability in a variety of fields—feature high in the ranking, thus reaffirming the complex nature of the underlying capabilities that are needed for their design and creation.

### 3.2. The Most Complex Green Technologies and the Main Innovators

**Figure 4:**
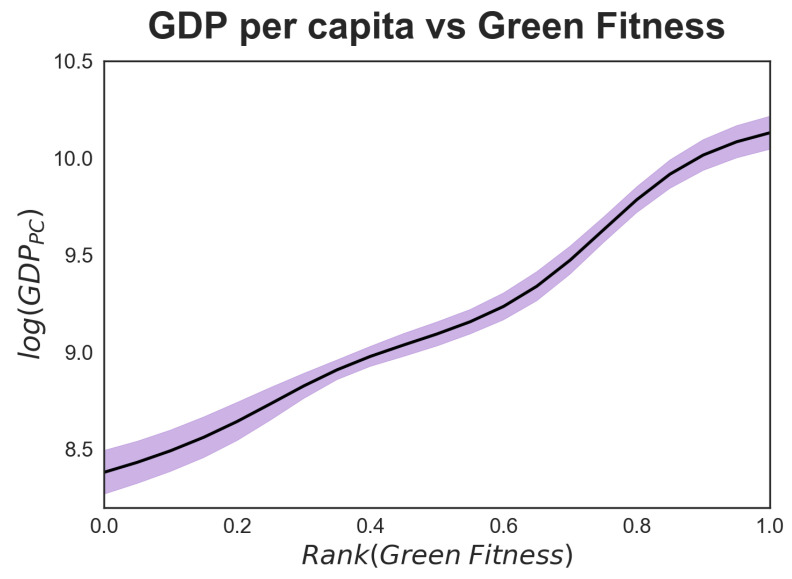
Correlation between green fitness ranking and per capita GDP over the time interval 1980–2010. Green fitness, as a proxy for the green innovative capacity of countries, is positively correlated with income per capita. The figure is obtained by pooling countries and years in our database. The expected value of green fitness is obtained through a non-parametric kernel estimation (black line), while the 95% confidence interval of the expected value (purple shadow) is computed with bootstrap.

Let us now juxtapose the information gathered so far and look into combined country-green technology patterns. In Figure 4 we plot the green fitness based on the country-green technology matrices M(y,10) against per capita GDP(y), for y∈[1980,2010]. By pooling all countries and years in our database, we estimate the expected value of green fitness through a non-parametric Nadaraya-Watson estimation with a Gaussian kernel [33]. The corresponding 95% confidence interval is computed with a bootstrap resampling. Figure 4 provides a generalization of what has emerged so far, namely that there is a positive relationship between average GDP per capita and our measure of green fitness. We opt for GDP as a proxy of living standards in a country for two reasons. The first is that GDP is a gold standard which helps us ground our exploratory study on green innovation better within the existing literature, primarily prior studies that use EFC approach on trade. For all the known limitations that GDP carries it remains the most widely used measure. The second reason is that when we contemplated the Human Development Index (HDI) [34] as an alternative (and more comprehensive) measure, we found a strong correlation with GDP and, thus, that findings were substantially unaltered.

**Figure 5:**
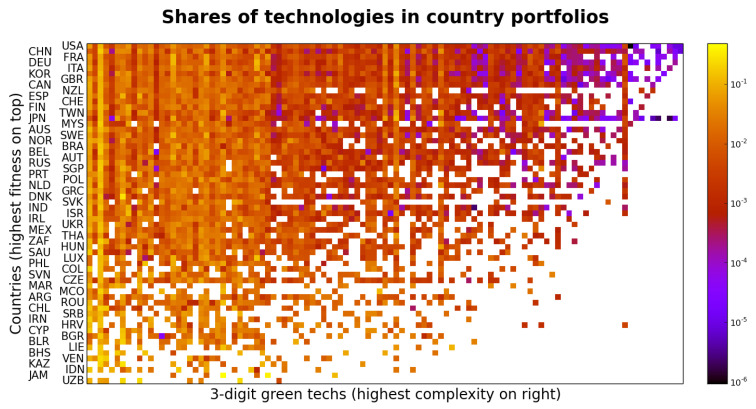
3-digit M(2010,10) with rows and columns ordered by green fitness and green complexity respectively. Colour represents the share of each technology within the technology basket of each country. The matrix shows a semi-triangular shape, accordingly to the EFC narrative, the highest green fitness countries are competitive in almost all technologies, from the most to the least complex, while the basket of technologies of lower fitness countries is limited to less complex technologies.

In turn, the triangular shape of the country-technology matrix of Figure 5 indicates that countries with higher levels of GDP per capita possess, as several scholars advocate, more developed capabilities that allow them to be major producers of more complex green technologies. By the same token, inventive efforts in poorer countries are limited to less complex technologies as a reflection of overall lower capabilities. These two snapshots confirm that the distribution of inventive capacity in green technology is broadly in line with prior literature [17,20,35].

Looking more in detail, Table 1 shows the ten most complex green technologies over the entire time period of the analysis (1971–2010) and, for each one, it lists the top five inventor countries, the share in total world green innovation and the corresponding RCA index. A few features emerge from this table. First, eight out of ten of the most complex technologies are for Climate Change Mitigation—the only two exceptions being GHG Capture and Storage, and Environmental Management. Second, in the upper part of the list are three types of enabling technologies, which indicates that the most advanced inventive efforts are currently devoted to perfecting existing technologies for wide, cross-sectoral purposes. Third, and related to the former, the list provides a balanced mix between mature technologies (i.e., enabling or nuclear energy) and very experimental ones (i.e., carbon capture, superconducting elements for efficient energy distribution). Fourth, the table also portrays a balanced picture as the key environmental priorities encompass areas like transport, waste, industrial production, energy and buildings. Fifth, as already anticipated earlier, the leading producers are all high-income countries. Another notable feature is the recurrence of Asian catching-up countries in various domains. South Korea ranks high in all but two (i.e., environmental management and rail transport) as a reflection of the environmental challenges due to a wide industrial mix (e.g., [36]). China excels in waste management, rail transport, industrial production and energy, a profile that resonates with the heterogeneity of emission sources due to remarkable regional and sectoral differences [37]. Conversely, Taiwan only appears in waste management, plausibly as a result of targeted policy efforts (e.g., [38]). Table 2 reports the same information as Table 1, but for the lower-complexity technologies. Unsurprisingly, also in this case the top 5 innovators per technology are high-fitness countries, which consistently with the triangular structure of Figure 5, have the necessary capabilities to excel across the spectrum, while low-fitness countries perform relatively well only in mundane technologies.

**Figure 6:**
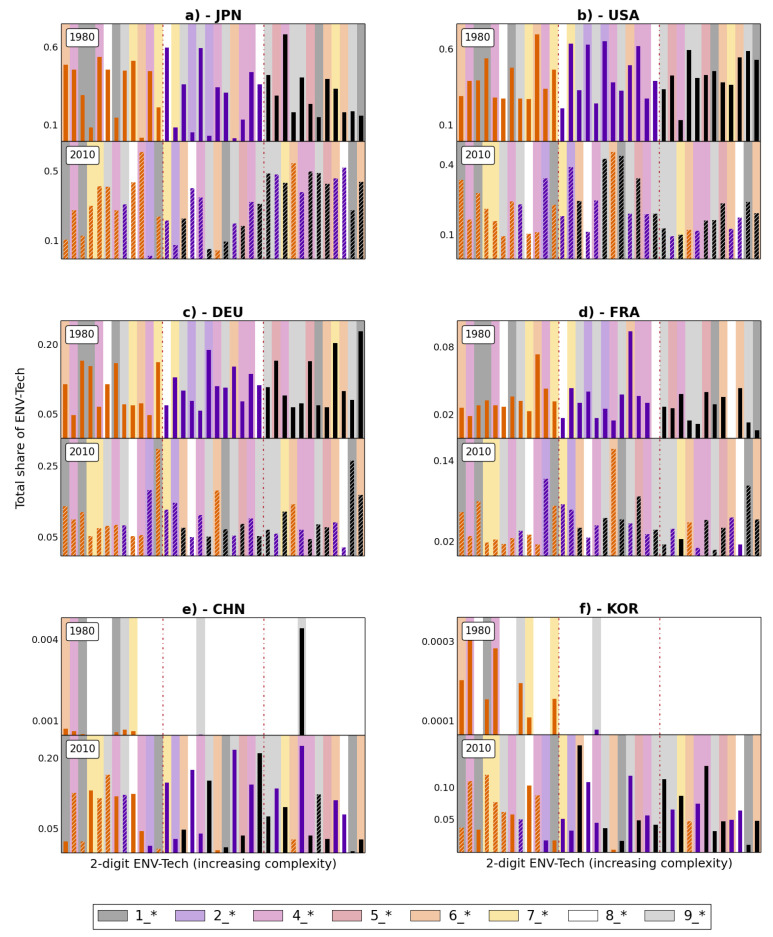
Composition of national green technology baskets. Each panel illustrates the share of patents produced by a selection of countries in each 2-digit technological field in 1980 (**upper part**) and 2010 (**bottom**). Technologies are ordered by increasing complexity. The colour of the bars indicates the ranking of each technology in 1980, while the background colour stands for the 1-digit technology to which each bar belongs (see list on p. 3). The hatched pattern is for technologies that are observed in both time windows.

Figure 6 focuses on a sample of top countries as of 2010 and shows that there is heterogeneity in the composition of the portfolios of such top innovators. Therein each panel contains the shares of patenting in all green technologies (ordered by increasing green complexity from left to right) in the first and final decade. For instance, Japan is relatively focused on the the most complex technologies. This contrasts with the country profiles of, say, the US or France which instead have a more balanced portfolio of green innovation across the complexity spectrum. The above is informative of the differential contribution of countries to the advancement of the green technology frontier. Moreover, this broad and long-term view allows us to discern countries that have been leaders since the beginning of the period, such as Japan, the US, France and Germany, from the latecomers like China and South Korea, which indeed only started to patent in the 1990s. The distribution of the patenting shares for each country-decade panel reveals the direction of inventive efforts. For instance, in the last decade Japan (Panel A of Figure 6) stands out as rather proactive in complex technologies with high and low complexity, rather than those in the middle. By contrast, the relative contribution of the US (Panel B of Figure 6) has decreased after the 1990s, due to the entry of other actors, as highlighted earlier in regards to Figure 1. In relative terms, and compared to Japan, the distribution of US shares in green technology is higher in technologies with middle levels of complexity. The relative shares of Germany and France (Panels C and D of Figure 6) are somewhat constant over time and spread evenly across the whole technological spectrum. Interestingly, newcomers like China and South Korea (Panels E and F of Figure 6) join the global path of green technology innovation with contributions to both less and more complex technologies.

### 3.3. How Does Green Innovation Capacity Vary with Income and Trade?

**Figure 7:**
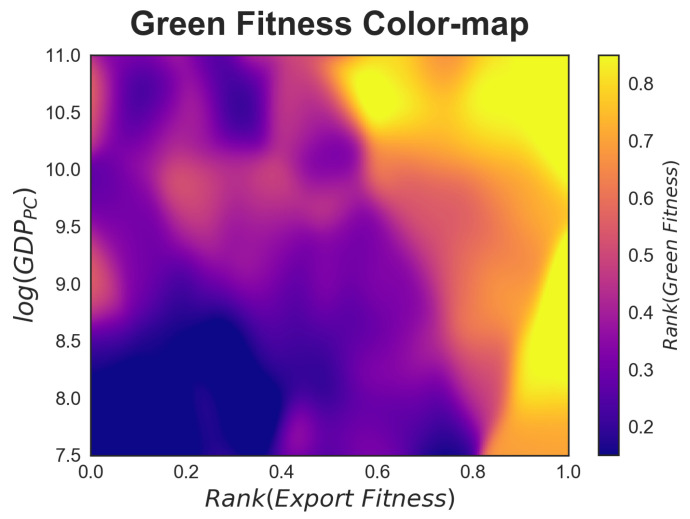
The three-dimensional relation between export fitness, GDP per capita, and green fitness. The colour map represents the variation of green fitness obtained with a non-parametric Nadaraya-Watson kernel estimation by pooling all countries in our database over the time interval 1980–2010.

**Figure A4:**
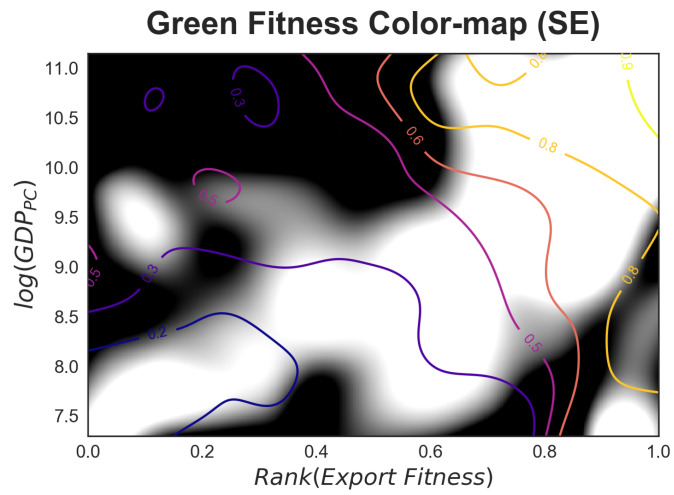
Estimation error of the green fitness colour map in Figure 7. The plot is built with the same data of Figure 7. Two layers of information are represented in this figure. (1) In the grey scale, the green fitness ranking estimation error in the Nadaraya-Watson kernel method. White indicates a standard error of ∼0.2% or less, and black a standard error of ∼0.4% or more. (2) The iso-lines of the green fitness ranking levels (lowest in deep purple, highest in clear yellow). The plot is obtained by pooling all countries in our database over the time interval 1980–2010. The different shades of black and white confirm our findings: export fitness and GDP per capita are complementary in determining the green technological capabilities of countries.

Coherent with the argument that the accumulation of competences is a vehicle for fostering growth [39], Figure 4 in the previous subsection hints at a strong positive correlation between green innovation and per capita income. At the same time, Figure 2 highlights a divide between mid-ranking countries, whereby some manage to climb up the green technology complexity ladder (i.e., China and South Korea) while others do not (i.e., Argentina, Bulgaria). No doubt, the structural characteristics of a country play a fundamental role in unleashing the innovation potential, and in this part of the paper we investigate some of these characteristics and the extent of their impulse. Given the exploratory nature of our analysis, in Figure 7 we focus on GDP per capita (as a proxy of standards of living and economic growth potential in each country) and export fitness (as a proxy of the trade performance of each country) [40]. We propose a graphical analysis based on a colour map which portrays the relation between GDP per capita and export fitness on the *x*-*y* axes, and the entire range of green technological fitness for all the countries in our database on the *z*-axis, represented with colour variation. In this case, as for Figure 4, green fitness is computed for each year as a moving average over a δ=10. The colour map is obtained through a 3-dimensional Nadaraya-Watson non-parametric estimation [33] fed with a pooling of all countries in our database over the period 1980–2010. Figure A4 in Appendix E provides information about the green fitness estimation error, for ease of comparability with Figure 7 the iso-levels of green fitness are superimposed on the plot. Notice that the confidence level of the Nadaraya-Watson estimation is heterogeneously distributed in the export fitness-GDP per capita plane.

The areas of Figure 7 with higher intensity are those of greater interest. The purple-coloured portion at the bottom left-hand of the graph indicates that, as expected, countries with low GDP and low export fitness exhibit the lowest green technology fitness; also expected is the growth of green fitness as one moves towards the top right corner of the plot.

Another interesting portion of this diagram is on the right-hand side, where intermediate levels of (log) GDP per capita (between 8 and 9.5) and very high export fitness correspond to very high levels of green fitness. This indicates that a highly diversified portfolio of trade matters for unleashing innovation capacity among both high- and mid-level income countries. Put otherwise, a country’s level of wealth is not a barrier to developing advanced competences for environmental innovation insofar as they engage trade of more complex products. The diagonal movement of colour is in agreement with the EFC narrative according to which countries with higher export fitness than per capita GDP show a level of complexity that has not yet translated into higher income, but indicates higher development and growth potential [35]. This finding resonates with the descriptive analysis of the rankings in the previous subsections, where the performance of emerging countries in green innovation has been commented on. It also resonates with the recombinant nature of the technology at hand, and the fact that green patents exhibit more diversity of technical components and of know-how relative to non-green ones [41]. Openness to trade and strategic specialization in key components for green technologies are thus likely to enable middle-income countries to accelerate in the pursuit of environmental innovation. This is especially true if we consider the high levels of fitness of enabling technologies that bring together different pools of know-how into coherent solutions for wide applicability.

## 4. Conclusions

This paper uses a Economic Fitness-Complexity approach to analyse green innovation trends across countries and technological fields over a forty year period. The main questions we have addressed are: which countries innovate the most? What are the most complex green technologies? What is the relationship between economic development and specialisation in environmental technologies?

We make three major contributions to the literature. First, we provide an overview of spatial and temporal characteristics of green innovation by exploiting the geo-localisation of patent data. Second, we move beyond aggregate trends and delve into the relative performance of each country in relation to the complexity of the technology. This allows us to identify three typologies of countries: leaders, followers, and laggards. As expected, there is a direct relationship between GDP per capita and innovation capacity. That said, we also observe the growing relevance of countries that started from behind but that managed to become prominent actors. Most of these are based in East Asia. Third, we complement previous studies on green technology with a deeper understanding of how innovation capacity is distributed across areas of specialisation. The fitness ranking approach reveals that, after a period of deeper specialisation within diverse domains, innovation in green technology has become more horizontal, with bigger efforts being observed in cross-domain, or enabling, technologies. This trend seems to indicate that while the relative stage of development of individual areas—such as i.e., renewable energy generation or waste management—may have peaked in terms of technology life cycle, there is now demand for greater interoperability across green technologies—i.e., the integration of Information and Communication Technologies for monitoring energy distribution. A combination of more general characteristics of economic performance, such as greater cost efficiency and openness to trade, may entail that opportunities exist for countries that have remained at the margin of the geo-politics of climate change adaptation and mitigation. We hope that the empirical findings of our exploratory study will encourage further analysis of the untapped development potential of environmental sustainability, especially for fast growing countries at the periphery.

## Figures and Tables

**Table 1:** Top innovators in the most complex green technologies.

Technology Family	Technology Group	Top 5 Innovators	Share	RCA
CCMT for transportation	Enabling Technologies 6.5 (example: Electric vehicle charging)	JPN	0.441	1.126
		USA	0.196	1.100
		DEU	0.172	1.543
		FRA	0.054	1.394
		KOR	0.049	0.676
Environmental management	Environmental Monitoring 1.5 (example: Tools for environmental data analysis)	JPN	0.279	0.713
		DEU	0.267	2.400
		USA	0.243	1.366
		FRA	0.104	2.706
		SWE	0.020	3.700
CCMT for wastewater treatment or waste management	Enabling Technologies 8.3 (example: Landfilling with gas recovery)	JPN	0.522	1.333
		USA	0.177	0.991
		CHN	0.082	1.039
		KOR	0.066	0.901
		TWN	0.037	1.980
CCMT for transportation	Rail Transport 6.2 (example: Reducing energy consumption)	JPN	0.461	1.176
		DEU	0.129	0.725
		USA	0.112	1.420
		FRA	0.094	0.847
		KOR	0.056	1.464
Capture, storage, sequestration, or disposal of GHGs	Capture or Disposal of Gases other than $CO_{2}$ 5.2 (example: Chemical nitrification inhibitors)	JPN	0.430	1.098
		USA	0.238	1.333
		DEU	0.080	0.720
		KOR	0.049	0.669
		FRA	0.041	1.068
CCMT for production or processing of goods	Enabling Technologies 9.8 (example: Direct digital manufacturing)	JPN	0.492	1.254
		USA	0.165	0.927
		CHN	0.124	1.585
		DEU	0.088	0.789
		KOR	0.033	0.458
CCMT for energy generation, transmission or distribution	Nuclear Energy 4.4 (example: Nuclear fusion reactors)	JPN	0.501	1.277
		USA	0.163	0.915
		KOR	0.135	1.853
		FRA	0.053	1.373
		DEU	0.047	0.424
CCMT for energy generation, transmission or distribution	Technologies for Efficient Electrical Power Generation, Transmission or Distribution 4.5 (example: Superconducting electric elements or equipment)	JPN	0.384	0.979
		CHN	0.228	2.901
		USA	0.120	0.671
		KOR	0.076	1.048
		DEU	0.073	0.657
CCMT for transportation	Road Transport 6.1 (example: Hybrid vehicles)	JPN	0.548	1.397
		DEU	0.145	1.307
		USA	0.124	0.696
		FRA	0.049	1.284
		KOR	0.048	0.662
CCMT for buildings	Architectural or Constructional Elements Improving Thermal Performance 7.3 (example: Retrofit insulation)	JPN	0.437	1.114
		DEU	0.124	1.117
		USA	0.104	0.582
		CHN	0.098	1.242
		KOR	0.088	1.207

**Table 2:** Top innovators in the least complex green technologies.

Technology Family	Technology Group	Top 5 Innovators	Share	RCA
Environmental Management	Water Pollution Abatement 1.2 (example: Oil spill cleanup)	USA	0.338	1.899
		DEU	0.1340	1.255
		JPN	0.110	0.281
		FRA	0.065	1.681
		KOR	0.038	0.526
CCMT related to energy generation, transmission or distribution	Renewable Energy Generation 4.1 (example: Wind energy)	JPN	0.278	0.707
		USA	0.168	0.944
		CHN	0.127	1.616
		KOR	0.111	1.524
		DEU	0.102	0.920
Environmental Management	Waste Management 1.3 (example: Material recycling)	USA	0.281	1.58
		JPN	0.132	0.339
		DEU	0.122	1.110
		FRA	0.081	2.098
		ITA	0.050	4.199
CCMT for buildings	Energy Efficiency in Buildings 7.2 (example: Lighting)	JPN	0.303	0.773
		USA	0.213	1.197
		CHN	0.132	1.683
		KOR	0.121	1.661
		DEU	0.055	0.497
CCMT for buildings	Enabling Technologies in Buildings 7.4 (example: Enabling technologies or technologies with a potential or indirect contribution to GHG emissions mitigation)	JPN	0.418	1.066
		USA	0.162	0.911
		CHN	0.116	1.472
		KOR	0.079	1.080
		DEU	0.077	0.696
CCMT in the production or processing of goods	Technologies Related to Metal Processing 9.1 (example: Reduction of greenhouse gas [GHG] emissions)	JPN	0.412	1.052
		CHN	0.166	2.119
		USA	0.096	0.542
		DEU	0.084	0.754
		KOR	0.063	0.866
CCMT for energy generation, transmission or distribution	Energy Generation from Fuels of Non-Fossil Origin 4.2 (example: Biofuels)	JPN	0.279	0.7111
		USA	0.245	1.378
		CHN	0.120	1.526
		DEU	0.086	0.777
		KOR	0.059	0.815
CCMT in the production or processing of goods	Technologies Relating to Chemical Industry 9.2 (example: Improvements relating to chlorine production)	JPN	0.313	0.797
		USA	0.233	1.306
		CHN	0.123	1.572
		DEU	0.086	0.774
		KOR	0.0515	0.707
CCMT for wastewater treatment or waste management	Solid Waste Management 8.2 (example: Waste collection, transportation, transfer or storage)	JPN	0.439	1.121
		CHN	0.125	1.593
		USA	0.108	0.604
		KOR	0.104	1.434
		DEU	0.055	0.499
CCMT for energy generation, transmission or distribution	Enabling Technologies 4.6 (example: Energy storage)	JPN	0.614	1.566
		USA	0.113	0.635
		KOR	0.089	1.219
		DEU	0.058	0.520
		CHN	0.045	0.579

**Figure A1:**
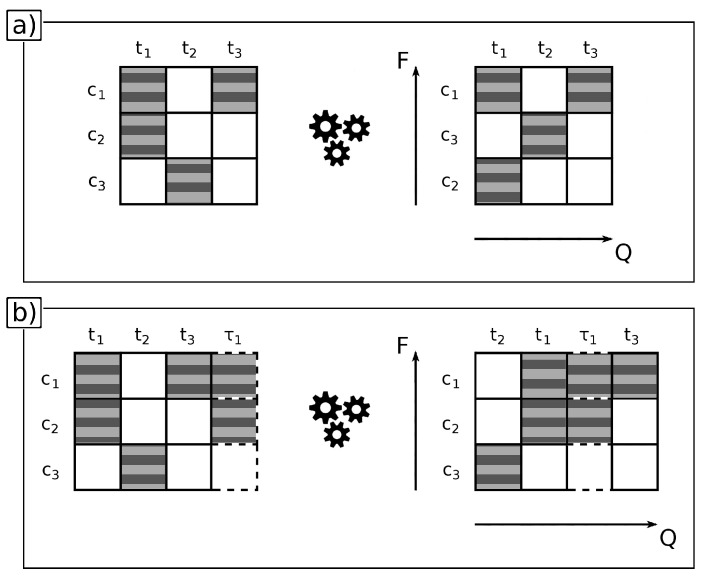
Panel a) depicts a binary country-technology matrix *M* consisting of three countries ($c_{1}$, $c_{2}$, and $c_{3}$) and three technologies ($t_{1}$, $t_{2}$, and $t_{3}$); ordering the rows by fitness and the columns by complexity (right), we see that $t_{1}$ is more complex than $t_{2}$ and $c_{3}$ has higher fitness than $c_{2}$. Panel b) depicts the same matrix *M* of panel a), to which an additional column $\tau_{1}$ has been added; ordering the rows by fitness and the columns by complexity (right), we see that now $t_{2}$ is more complex than $t_{1}$ and $c_{2}$ has higher fitness than $c_{3}$. The figure shows that, in general, the addition (or subtraction) of columns to M(y) can potentially affect fitness and complexity rankings.

**Figure A2:**
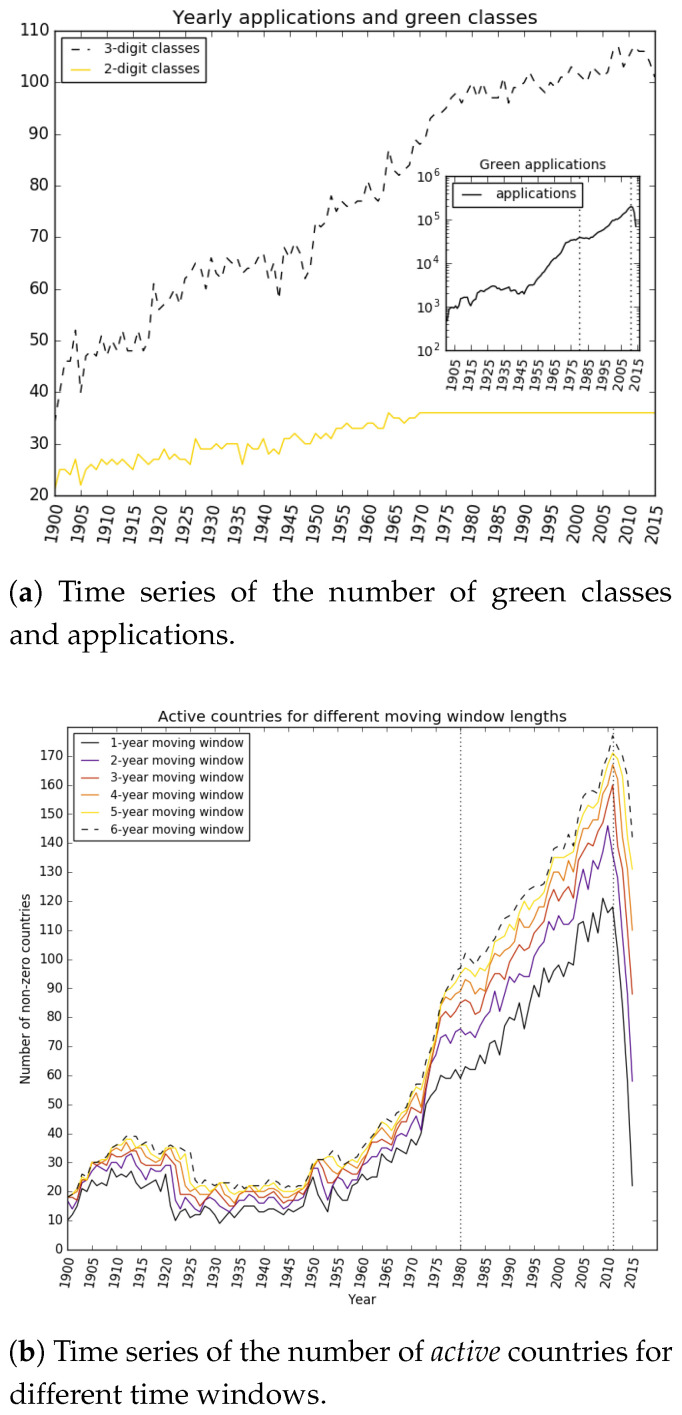
Yearly green classes, applications, and *active* countries.

**Figure A3:**
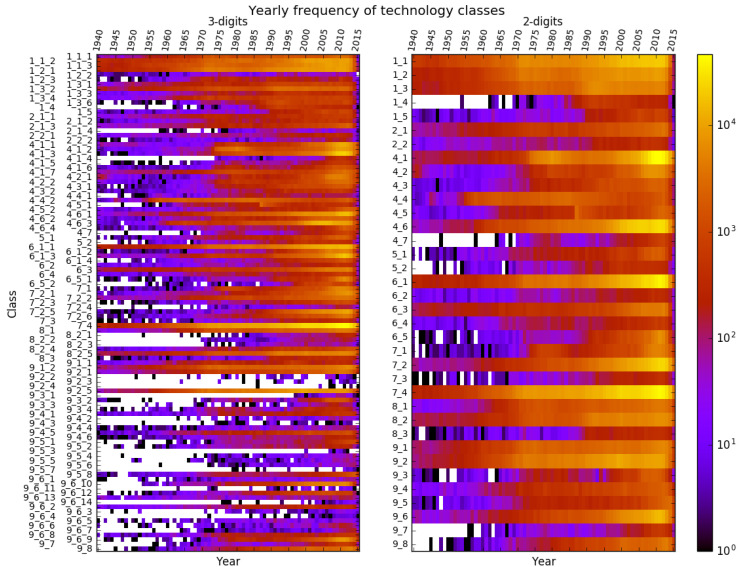
Yearly frequency of green technology classes (left:3-digits, right: 2-digits).

## Abbreviations

The following abbreviations are used in this manuscript:

GHG	Greenhouse Gases
EPO	European Patent Office
IPC	International Patent Classification
CPC	Cooperative Patent Classification
EFC	Economic Fitness-Complexity
GDP	Gross Domestic Product
CCMT	Climate-Change Mitigation Technology
OECD	Organisation for Economic Co-operation and Development

## Appendix A. Countries and Technologies: Codes and Descriptions

**Table A1:** 2-digit ENV-Tech codes and labels.

Code	1-Digit Class Description	2-Digit Class Description
1	Environmental Management	
1_1		Air pollution abatement
1_2		Water pollution abatement
1_3		Waste management
1_4		Soil remediation
1_5		Environmental monitoring
2	Water-related adaptation technologies	
2_1		Demand-side technologies (water conservation)
2_2		Supply side technologies (water availability)
4	CCMTs related to energy generation, transmission or distribution	
4_1		Renewable energy generation
4_2		Energy generation from fuels of non-fossil origin
4_3		Combustion technologies with mitigation potential (e.g., Using fossil fuels, biomass, waste, etc.)
4_4		Nuclear energy
4_5		Efficiency in electrical power generation, transmission or distribution
4_6		Enabling technologies in energy sector
4_7		Other energy conversion or management systems reducing ghg emissions
5	Capture, storage, sequestration or disposal of greenhouse gases	
5_1		$CO_{2}$ capture or storage (ccs)
5_2		Capture or disposal of greenhouse gases other than carbon dioxide ($N_{2}O$, $CH_{4}$, pfc, hfc, $SF_{6}$)
6	CCMTs related to transportation	
6_1		Road transport
6_2		Rail transport
6_3		Air transport
6_4		Maritime or waterways transport
6_5		Enabling technologies in transport
7	CCMTs related to buildings	
7_1		Integration of renewable energy sources in buildings
7_2		Energy efficiency in buildings
7_3		Architectural or constructional elements improving the thermal performance of buildings
7_4		Enabling technologies in buildings
8	CCMTs related to waste water treatment or waste management	
8_1		Wastewater treatment
8_2		Solid waste management
8_3		Enabling technologies or technologies with a potential or indirect contribution to ghg mitigation
9	CCMTs in the production or processing of goods	
9_1		Technologies related to metal processing
9_2		Technologies relating to chemical industry
9_3		Technologies relating to oil refining and petrochemical industry
9_4		Technologies relating to the processing of minerals
9_5		Technologies relating to agriculture, livestock or agroalimentary industries
9_6		Technologies in the production process for final industrial or consumer products
9_7		Climate change mitigation technologies for sector-wide applications
9_8		Enabling technologies with a potential contribution to ghg emissions mitigation

**Table A2:** ISO3 country codes and names.

ISO3 Code	Country Name	ISO3 Code	Country Name	ISO3 Code	Country Name
ARG	Argentina	GRC	Greece	NOR	Norway
AUS	Australia	HRV	Croatia	NZL	New Zealand
AUT	Austria	HUN	Hungary	PHL	Philippines
BEL	Belgium	IDN	Indonesia	POL	Poland
BGR	Bulgaria	IND	India	PRT	Portugal
BHS	Bahamas	IRL	Ireland	ROU	Romania
BLR	Belarus	IRN	Iran	RUS	Russian Federation
BRA	Brazil	ISR	Israel	SAU	Saudi Arabia
CAN	Canada	ITA	Italy	SGP	Singapore
CHE	Switzerland	JAM	Jamaica	SRB	Serbia
CHL	Chile	MAR	Morocco	SVK	Slovakia
CHN	China	MCO	Monaco	SVN	Slovenia
COL	Colombia	MEX	Mexico	SWE	Sweden
CYP	Cyprus	MYS	Malaysia	THA	Thailand
CZE	Czech Republic	NLD	Netherlands	TWN	Taiwan
DEU	Germany	JPN	Japan	UKR	Ukraine
DNK	Denmark	KAZ	Kazakhstan	USA	United States of America
ESP	Spain	KOR	South Korea	UZB	Uzbekistan
FIN	Finland	LIE	Liechtenstein	VEN	Venezuela
FRA	France	LUX	Luxembourg	ZAF	South Africa
GBR	United Kingdom				

## Appendix B. Column Selection and Technological Sector Fitness

In general, the fitness and complexity rankings produced by the EFC algorithm are not invariant to the addition (or subtraction) of rows or columns in the binary matrix. Figure A1 depicts a toy example illustrating this point. The right part of panel (**a**) depicts a 3-by-3 matrix M with rows and columns sorted by fitness and complexity respectively. In particular, row $c_{1}$ has higher fitness than $c_{3}$, which in turn has higher fitness than $c_{2}$; columns instead can be ordered in decreasing order of complexity as follows: $t_{1}$, $t_{2}$, $t_{3}$. Panel (**b**) represents the same matrix to which one additional column $\tau_{1}$ was added. Ordering the rows and columns of the new matrix with the EFC algorithm, both the ranking of rows and columns changes. In particular, $c_{2}$ is now fitter than $c_{3}$ and $t_{2}$ is more complex than $t_{1}$.

This example indicates that applying sector fitness to technologies might yield biased results. However, two remarks mitigate this concern. First, while the figure shows that it is in general possible to alter the ranking by simply adding a column, we had to choose a rather extreme case to make the point. In fact, column $\tau_{1}$ is built in such a way to bring very close together the compositions of the most and least fit row of panel (**a**). However, this is quite unrealistic because it would be much harder to achieve if the matrix were substantially larger (as are the empirical matrices of the analysis). On the other hand, adding $\tau_{1}$ is akin to adding adding the information that a country the was thought to patent only in a very ubiquitous agricultural field and nothing else, also patents in ground breaking medical technologies. This however looks intuitively implausible and contrasts with the evidence shown e.g., in Figure 5.

Finally, it is worth noting that, although it is true that applying sector fitness to green technologies cuts some potentially relevant columns from M, it is also the case that these omitted fields are not exclusively linked to green technologies, otherwise they would certainly be included in the classification. Hence, even if we were to include them, we would only account for them partially, and, since we know that they can potentially influence the fitness and complexity rankings, adding them to the analysis would not make us feel any safer about the eventual introduction of biases in the results of the analysis performed with the EFC algorithm. For this reason, we believe that omitting the columns that are not exclusively (or very predominantly) linked to green technologies should not be a major shortcoming.

## Appendix C. Measurement of National Knowledge Bases

The geographical coordinates of each inventor’s address were obtained through the GeoNames database [42], which contains worldwide geographical information on, among others, administrative borders and postal codes. We first try to detect a postal code in the address string and use GeoNames postal code information to assign geographical coordinates to it. For the remaining addresses, we try to identify the name of a city and use GeoNames city information to obtain the coordinates. Finally, for the addresses not geolocalised in the first two steps, we send the address to the Google Maps API which returns geographical coordinates. However, even if the EPO is improving and updating every year the Patstat database, an important share of inventor’s addresses is still missing. To deal with this issue, we exploit the work by the Institut Francilien Recherche Innovation Société (IFRIS) filling missing addresses with other patent databases i.e., REGPAT and National Patent Databases [43]. Although Patstat assigns an unambiguous identifier to each applicant or inventor, multiple IDs assigned to the same person could be retrieved. In these cases, information on the applicant/inventor address may be fully provided by some IDs and missing in others. Hence, to reduce the number of applicants/inventors with a missing address, we focus on patent families and analyse multiple IDs in order to detect non-missing address assigned to the same person. To do so, for each missing address we calculate the Levenshtein distance between the inventor name and each of the other names with a complete address. In those cases in which the indicator value is lower than three, we assign the address of the inventor found to complete the missing one [44]. In so doing, we obtain 799,011 (67.7%) green patent families with at least one inventor geo-localised, distributed among 141 countries. Patent families without geo-localised inventor (either because inventor information is missing or because address is not found) has been dropped from the dataset. Variations of the geo-localisation rate across ENV-TECH families and patent offices are very small: the standard deviation is 0.089 in the first case and 0.118 in the second case, when you take into account the top 10 patent offices (accounting for 93% of all the patent families), thus we can conclude that the bias introduced by dropping non geo-localised inventors is negligible.

## Appendix D. Data Aggregation: Possible Advantages

Figure A2 suggests that the length of the time window we choose to study and its extremes can have a significant effect on the composition of each snapshot of data. For instance, Figure A2a shows that the number of *active* [45] technology classes (Figure A2a) and active countries (Figure A2b) has grown considerably over time, which implies that a clear trade-off exists between the length of the time series and the size of the intersection of the data available in each year. This is also due to the fact that the the number of applications containing green technologies has remained quite small until the 1980s (Figure A2a, inset). Notice that the trade-off is quite sharp also if we move the right extreme of the time interval too far forward, since there is a sharp drop in the number of filed patent applications, and hence in the density of the data matrices after 2012. This is compatible with the presence of a constant backlog of applications that have been filed but not yet examined and, for this reason, not yet added to the databases. The delay between filing and inclusion into Patstat varies depending on the patent office that received the application. For example, the backlog at the United States Patent and Trademark Office (USPTO) is estimated at around 40 months on average; this implies that the data for 2012 contained in the 2016a edition of the database is most likely still incomplete.

Figure A2 shows that by averaging over a longer time window (δ) and counting the number of non-zero rows in the average weighed matrix the upper bound on the number of potentially active countries in matrix $M^{\delta }\left(y\right)$ defined by applying Equation (1) to Equation (4) increases substantially, especially since the mid 1970s. The further advantage of averaging over a reasonably long time window is that it would also help get rid of small “holes” in the presence of some technology classes, especially at the 2-digit level, as shown in figure the right panel of Figure A3, which counts the frequency of each 2-digit class within every $M^{1}\left(y\right)$ over the period 1940–2015. The left panel, which reports the yearly count of 3-digit classes, clearly shows that, at this disaggregation, some classes are too recent and inconsistently represented in the data to be included into an inter-temporal comparison.

## Appendix E. The Relationship between Export Fitness, GDP Per Capita and Green Fitness: Estimation Error

To validate the three-dimensional analysis of the relationship between export fitness, GDP per capita and green fitness represented as a colour map in Figure 7, we compute the standard error (SE) of the green fitness Nadaraya-Watson estimation. As can be observed in Figure A4, where the portions of the plot with SE≳0.4% are in black, and those with SE≲0.2% are in white, the standard error is very heterogeneous. In fact, since GDP per capita and export fitness are positively correlated [35,46], most of the points used in our estimation lie on the diagonal of the x-y plane. Only a few countries show different trends, for instance China has higher export fitness than per capita GDP and lies in the bottom-left corner of the graph, by contrast most of the oil exporters have higher income than export fitness, and thus they are placed in the opposite corner. The diagonal shades of grey and white are consistent with our narrative. The green technological competitiveness of countries, proxied by green fitness, is determined by the interplay of export fitness and GDP per capita, and the role of export fitness can compensate low levels of income per capita.

## References

[1] Deacon R.T., Mueller B., Ramón López M., Toman M. (2006). Political economy and natural resource use. Economic Development and Environmental Sustainability: New Policy Options.

[2] Romer P.M. (1994). The origins of endogenous growth. J. Econ. Perspect..

[3] Weitzman M.L. (1998). Recombinant growth. Q. J. Econ..

[4] Cohen W., Levinthal D.A. (1990). Absorptive capacity: A new perspective on learning and innovation. Adm. Sci. Q..

[5] Atkinson A.B., Stiglitz J.E. (1969). A new view of technological change. Econ. J..

[6] Chatterjee S., Wernerfelt B. (1991). The link between resources and type of diversification: Theory and evidence. Strateg. Manag. J..

[7] Napolitano L., Evangelou E., Pugliese E., Zeppini P., Room G. (2018). Technology networks: The autocatalytic origins of innovation. R. Soc. Open Sci..

[8] Rigby D.L., Essletzbichler J. (1997). Evolution, process variety, and regional trajectories of technological change in US manufacturing. Econ. Geogr..

[9] Capello R., Nijkamp P. (2010). Handbook of Regional Growth and Development Theories.

[10] Kogut B., Zander U. (1993). Knowledge of the firm and the evolutionary theory of the multinational corporation. J. Int. Bus. Stud..

[11] Frenken K., Boschma R.A. (2007). A theoretical framework for evolutionary economic geography: Industrial dynamics and urban growth as a branching process. J. Econ. Geogr..

[12] Neffke F., Henning M., Boschma R. (2011). How do regions diversify over time? Industry relatedness and the development of new growth paths in regions. Econ. Geogr..

[13] Hidalgo C.A., Klinger B., Barabási A.L., Hausmann R. (2007). The product space conditions the development of nations. Science.

[14] Zaccaria A., Cristelli M., Tacchella A., Pietronero L. (2014). How the taxonomy of products drives the economic development of countries. PLoS ONE.

[15] Vona F., Consoli D. (2015). Innovation and skill dynamics: A life-cycle approach. Ind. Corp. Chang..

[16] Balland P.A., Rigby D. (2017). The geography of complex knowledge. Econ. Geogr..

[17] Tacchella A., Cristelli M., Caldarelli G., Gabrielli A., Pietronero L. (2012). A new metrics for countries’ fitness and products’ complexity. Sci. Rep..

[18] Cristelli M., Tacchella A., Cader M., Roster K., Pietronero L. (2017). On the Predictability of Growth.

[19] Tacchella A., Mazzilli D., Pietronero L. (2018). A dynamical systems approach to gross domestic product forecasting. Nat. Phys..

[20] Sbardella A., Pugliese E., Pietronero L. (2017). Economic development and wage inequality: A complex system analysis. PLoS ONE.

[21] Pugliese E., Cimini G., Patelli A., Zaccaria A., Pietronero L., Gabrielli A. (2017). Unfolding the innovation system for the development of countries: co-evolution of Science, Technology and Production. arXiv.

[22] Haščič I., Migotto M. (2015). Measuring Environmental Innovation Using Patent Data.

[23] Griliches Z. (1998). Patent statistics as economic indicators: A survey. R&D and Productivity: The Econometric Evidence.

[24] Lanjouw J.O., Pakes A., Putnam J. (1998). How to count patents and value intellectual property: The uses of patent renewal and application data. J. Ind. Econ..

[25] Arts S., Appio F.P., Van Looy B. (2013). Inventions shaping technological trajectories: Do existing patent indicators provide a comprehensive picture?. Scientometrics.

[26] EPO (2016). EPO Worldwide Patent Statistical Database Data Catalog.

[B27-entropy-20-00776] 27.For instance, at the lowest level of this hierarchy codes are very specific and refer to narrow technological fields, e.g., IPC full-digit C03C 1/02—“Pre-treated ingredients generally applicable to manufacture of glasses, glazes or vitreous enamels”. At the highest level, i.e., 1-digit, codes refer to general, broad technological domains, e.g., IPC 1-digit C—“Chemistry, Metallurgy”.

[28] Hall B.H., Helmers C. (2013). Innovation and diffusion of clean/green technology: Can patent commons help?. J. Environ. Econ. Manag..

[29] Balassa B. (1965). Trade liberalisation and “revealed” comparative advantage. Manch. Sch..

[B30-entropy-20-00776] 30.We do not expect the binarization strategy to have crucial effects on the results, especially if the matrices are sparse to begin with.

[31] Pugliese E., Zaccaria A., Pietronero L. (2016). On the convergence of the Fitness-Complexity Algorithm. Eur. Phys. J. Spec. Top..

[B32-entropy-20-00776] 32.This entails focusing on 36 technological categories rather than 94.

[33] Nadaraya E.A. (1964). On estimating regression. Theory Probab. Appl..

[B34-entropy-20-00776] 34.The HDI is a composite statistics developed within the United Nations Development Program to account for the intersection of three dimensions of a country well-being: health (proxied by life expectancy), education (mean years of schooling), and per capita income.

[35] Pugliese E., Chiarotti G.L., Zaccaria A., Pietronero L. (2017). Complex economies have a lateral escape from the poverty trap. PLoS ONE.

[36] Oh I., Wehrmeyer W., Mulugetta Y. (2010). Decomposition analysis and mitigation strategies of CO_2_ emissions from energy consumption in South Korea. Energy Policy.

[37] Liu Z., Geng Y., Lindner S., Guan D. (2012). Uncovering China’s greenhouse gas emission from regional and sectoral perspectives. Energy.

[38] Yang H.L., Innes R. (2007). Economic incentives and residential waste management in Taiwan: An empirical investigation. Environ. Resour. Econ..

[39] Nelson R.R., Phelps E.S. (1966). Investment in humans, technological diffusion, and economic growth. Am. Econ. Rev..

[B40-entropy-20-00776] 40.This is the sum of the complexities of the products exported by each country [17]. The economic fitness measure based on export data from the UN-COMTRADE database (available online at http://comtrade.un.org (accessed in January 2017)) is available for the period 1995–2015 at https://datacatalog.worldbank.org/dataset/economic-fitness (accessed in January 2017). However, in the present paper we employ the most recent version of the export fitness database, provided to us by the PIL group of the *Institute of Complex Systems-CNR* in Rome.

[41] Barbieri N., Marzucchi A., Rizzo U. (2018). Knowledge Sources and Impacts on Subsequent Inventions: Do Green Technologies Differ from Non-Green Ones?. https://papers.ssrn.com/sol3/papers.cfm?abstract_id=3164197.

[B42-entropy-20-00776] 42.GeoNames is a geographical database available under a Creative Commons attribution license, which contains over 10 million geographical names corresponding to over 9 million unique features whereof 2.8 million populated places and 5.5 million alternate names. A feature can be physical (e.g., mountain, lake), political (e.g., country, territory), a human settlement (e.g., city, village) etc. GeoNames is available online at http://www.geonames.org for further information (accessed on 12 May 2017).

[B43-entropy-20-00776] 43.For more details, check the repository available at https://github.com/cortext/Patstat.

[B44-entropy-20-00776] 44.For example, we can find in the same patent family two inventors with different IDs, the first one with a complete address, the second one with a missing one: “Gehri, Martin Christian Adrian” and “GEHRI, MARTIN, CHRISTIAN, ADRIAN”. As the Levenshtein distance between the two names is less than 3 when both strings are converted to upper case, we assume it is the same person and we use the complete address to fill the missing one.

[B45-entropy-20-00776] 45.We say that an entity (country or technology class) is active in a given time period if the sum of the application (or family) shares that refer to it is positive.

[46] Cristelli M., Tacchella A., Pietronero L. (2015). The heterogeneous dynamics of economic complexity. PLoS ONE.

